# Exploring Emotional Safety and Harm Among Hospitalized Patients: A Qualitative Study of Patients’ and Providers’ Perspectives

**DOI:** 10.3390/healthcare13151842

**Published:** 2025-07-29

**Authors:** Afsha Khan, Dildar Muhammad, Najma Naz, Sabiha Khanum, Awal Khan

**Affiliations:** 1 Institute of Nursing Sciences, Khyber Medical University, Peshawar 25100, Pakistan; dildar.ins@kmu.edu.pk (D.M.); najma.ins@kmu.edu.pk (N.N.); sabiha.ins@kmu.edu.pk (S.K.); 2 Medical Teaching Institution, Hayatabad Medical Complex, Peshawar 25110, Pakistan; awalkhanmsn@gmail.com

**Keywords:** emotional safety, hospitalization, patient experience, healthcare provider

## Abstract

**Background:** Emotional safety is increasingly recognized as crucial for high-quality patient care, encompassing a patient’s sense of security, courteous treatment, being heard, and a peaceful environment. **Purpose:** The purpose of this study was to explore the perceptions of patients and providers (doctors and nurses) regarding emotional harm and safety in relation to hospitalized patients. **Methods:** We conducted a qualitative study in public-sector teaching hospitals in Peshawar, Pakistan. Data were collected after we obtained informed consent using individual interviews with 15 providers, namely, doctors (n = 7) and nurses (n = 8), and five focus group discussions (FGDs) with 25 hospitalized patients. Data from both the interviews and FGDs were analyzed using Braun and Clarke’s six-phase approach to thematic analysis. **Results:** The key themes revealed by the providers’ perspectives were factors contributing to emotional harm, staff-related factors, coping mechanisms and solutions, and the impact of prior experiences and involvement. The main themes that emerged from the patients’ perspectives were anxiety upon admission, the impact of communication, emotional stress due to treatment delays, systemic/bureaucratic challenges, financial burden, a lack of emotional support, and post-hospitalization concerns. The consistent perspectives shared by both patients and providers included the impact of systemic factors, communication issues, the role of staff attitude/behavior, financial concerns, and the influence of prior experiences. **Conclusions:** This study highlights the complex interplay of systemic, staff-related, and patient-specific factors. It suggests a need to improve communication, staff support, administrative processes, financial counseling, emotional support integration, and discharge planning to minimize harm and create a patient-centered environment.

## 1. Introduction

Patient safety is an important factor in patient-centered care, where, alongside the traditional emphasis on physical safety, increasing attention is being paid to nonphysical aspects, including emotional and psychological factors [1]. Patient safety has conventionally been perceived as a professional and technological concern. Nonetheless, when safety is examined from patients’ perspectives, it is redefined as contingent, dynamic, and contestable [2]. Patients define safety in terms of “feeling safe,” which runs counter to the clinical definition of safety, which is measured in terms of clinical risk [1,3]. Patients are a significant yet overlooked source of knowledge [4]. Patient safety mainly concerns reducing physical and emotional harm experienced by patients, issues that may be more prevalent than once thought [5]. Patient views are frequently disregarded as emotionally unstable, illogical, or unreliable because of the epistemic power held by organizations and the practitioners working for them [4]. A hospital environment where patients feel safe fosters recovery, while poor quality of care makes patients feel unsafe, exerting both physical and psychological impacts, leading to poor patient recovery [6]. Furthermore, dealing with patients experiencing negative emotions such as anger, irritation, and frustration can elicit negative emotional states among healthcare providers, resulting in poor clinical decision-making by healthcare providers, thereby compromising patient safety [7]. Patients may feel unsafe not only during the occurrence of an error but also when the overall quality of their care is poor [6]. Few studies have specifically investigated nonphysical aspects such as the emotional safety of hospitalized patients. Owing to growing awareness, there have been calls for more research to determine the overall improvements in patient safety achieved so far [3,8]. Despite the growing recognition of emotional safety, its use and definition within the context of hospitalization are still ambiguous [1,9]. Although few studies have explored the concept of emotional safety and its role in patient care, highlighting patient vulnerability and power imbalances in healthcare, most existing research has discussed it as part of broader patient safety concerns. There is still a lack of detailed and context-specific understanding of emotional harm, especially from the combined perspectives of both patients and healthcare providers within the same healthcare system [2,4,10]. The existing evidence might not adequately represent patients’ subjective experiences and perceptions, so knowledge of their perspectives on safety is needed [1]. Implementing successful therapies to improve patient outcomes and care quality requires an awareness of patients’ perceptions of emotional safety and the exploration of the challenges patients face in attaining emotional safety, and meeting these requirements will ultimately enable medical staff to face their own challenges in this regard [1,3]. A new patient safety paradigm is essential because the traditional focus on merely minimizing physical harm and preventing errors, while important, is insufficient. This emerging paradigm advocates for a fundamental transformation that integrates patients’ comprehensive experiences of safety in the medical environment, moving beyond just “being safe” to encompass “feeling safe”. It is also equally important to explore healthcare providers’ perceptions, as their attitudes and behaviors heavily influence patients’ psychological safety [11,12]. The purpose of this qualitative study was to comprehensively explore emotional safety and emotional harm among hospitalized patients by investigating the emotional experiences of the patients alongside the perspectives of healthcare providers, specifically doctors and nurses. Unlike previous research, we aimed to identify and understand the various factors that influence patients’ emotional safety, thereby providing insights that can enhance healthcare practices and regulations and facilitate a more patient-centered approach.

## 2. Methodology

### 2.1. Study Design

In this qualitative study investigating emotional safety and harm among hospitalized patients, we integrated data collected independently from two distinct participant groups, namely, hospitalized patients and healthcare providers (doctors and nurses), to offer a comprehensive understanding of these factors from multiple perspectives within the same healthcare system. This study was conducted in accordance with the Consolidated Criteria for Reporting Qualitative Research (COREQ) principles/guidelines to ensure rigor and transparency.

### 2.2. Study Setting

This study was conducted in public-sector teaching hospitals in Peshawar, Khyber Pakhtunkhwa (KP), Pakistan. These are major tertiary care facilities chosen for their varied patient populations, multidisciplinary personnel, high patient flows, diverse mix of cases, and role in providing research and healthcare training, making them suitable settings for exploring emotional safety and harm.

### 2.3. Study Participants and Sampling

Data were collected from 25 patients divided into 5 focus groups and from 15 healthcare providers, namely, doctors (n = 7) and nurses (n = 8). A purposive sampling strategy with maximum variation was used to recruit participants. Maximum variation was achieved by intentionally selecting participants with diverse characteristics, including with respect to age, gender, educational background, and unit of admission or practice (e.g., medical, surgical, orthopedic, obstetrics, oncology, or psychiatry), as well as length of hospital stay (for patients) and years of experience (for providers).

To address the patient perspective, participants were recruited from various units to capture insights related to emotional safety during hospitalization. The inclusion criterion was admission for at least one week in one of the selected units. Patients exhibiting physical or mental deterioration—such that they were unable to communicate effectively, concentrate for the duration of a group discussion, or provide informed consent—were excluded to ensure their safety and the integrity of the data collected. Patients less than 18 years old or illiterate (i.e., unable to read and write) were also excluded.

To address provider perspectives, participants were recruited from several units to acquire a range of insights. Doctors and nurses who had worked for at least one year in these units were included. Interns, house officers, and those not directly involved in patient care (such as administrative staff) were excluded. Data collection continued until saturation was achieved.

Participants from various hospital units were recruited to understand emotional safety perspectives, considering unit-specific factors like patient acuity and staff workload. This heterogeneity may have introduced variability in the responses.

### 2.4. Data Collection

Data were collected using focus group discussions (held with patients) and individual interviews (with doctors and nurses). Focus group meetings were held with patients to encourage shared reflection and collective discussion of emotional safety experiences during hospitalization. In our study, the patients felt more comfortable speaking in groups, often validating and building upon each other’s experiences, enriching the data. In contrast, individual interviews were conducted with healthcare providers to promote candid and detailed responses regarding sensitive clinical practice issues, which otherwise might not have been openly discussed in a group setting. The duration of FGDs ranged from 45 to 60 min. FGDs were held in private rooms within the medical, surgical, and orthopedic units at the participants’ agreed-upon times to avoid disrupting their care. Recruitment was facilitated through nursing staff who initially informed eligible patients about the study. Interested patients were then approached individually by a member of the research team to provide detailed information. For doctors and nurses, individual interviews were conducted. The interviews took place at the participants’ agreed-upon times and locations during their breaks within the hospital premises. The duration of each interview ranged from 35 to 60 min. While some patients and healthcare providers were recruited from the same units, data collection was conducted separately and confidentially. All focus groups and interviews were held in private settings, and no identifiable information was shared across participant groups. Measures were taken to ensure that patients and providers were unaware of each other’s participation in the study. For both focus group discussions and individual interviews, open-ended questions based on an interview guide (see Supplementary Materials) were asked, covering thoughts on emotional harm, emotional safety, aspects making hospitalized patients feel emotionally unsafe, and suggested improvements. The interview guide was validated by a healthcare professional with expertise in patient safety and clinical psychology. FGDs and interviews were held in the Urdu language, recorded digitally, and supplemented by field notes.

### 2.5. Data Analysis

For analysis, Braun and Clarke’s six-phase approach of thematic analysis was used for both sets of data because of its flexibility and suitability for exploring participants’ subjective experiences in depth. The process involved data familiarization, initial code generation, theme identification, review, refinement, and final report production, allowing inductive pattern identification and systematic organization of emotional safety perceptions. Interview recordings (concerning the providers) and FGD recordings (regarding the patients) were initially transcribed into Urdu and later translated into English. To ensure the accuracy of translation from Urdu to English, the transcripts were first translated by a bilingual researcher and then back-translated by an independent expert to check for consistency and meaning. The transcripts were repeatedly read to ensure familiarity with the data. The translated transcripts were repeatedly checked for accuracy, and expert validation was sought to improve trustworthiness. Initial codes were assigned to relevant words and sentences through open coding. Similar codes were arranged into categories. Coding was independently and manually performed by two researchers, and any disagreement was discussed until a consensus was reached. Categories were further refined into distinct themes to prevent overlap. Member checking was conducted with the study participants to validate interpretations after preliminary theme development. Direct participant quotes were included to provide context and foster richness.

In this study, all five researchers had experience in qualitative research, with the primary researcher also having experience in patient safety research. None of the members of the research team had any prior relationships with the study participants. The researchers maintained reflexivity by taking field notes and engaging in discussions to avoid personal influence. The field notes were reviewed alongside the transcripts and used to facilitate contextual understanding, clarify non-verbal cues, and support the interpretation of emerging themes during the coding and thematic analysis process.

### 2.6. Ethical Consideration

Ethical approval for this study was granted by the Ethical Review Board of Khyber Medical University, Peshawar, Pakistan. Written informed consent forms were signed by all the participants after full disclosure of information before data collection commenced. The participants’ queries were explicitly answered before data collection.

## 3. Results

Table 1 shows the demographic characteristics of the study participants (both patients and providers).

### 3.1. Patients’ Perspectives

Table 2, the findings from the FGDs held with hospitalized patients revealed rich insights into the emotional safety of hospitalized patients. Seven main themes emerged, each expressing an important aspect of patient experiences. Among these themes were anxiety upon admission, the impact of communication, emotional stress due to treatment delays, systemic challenges, financial burden, lack of emotional support, and post-hospitalization worries.

#### 3.1.1. Pre-Hospital Anxiety

Many of the study participants were quite worried before and during the admission procedure. They exhibited anxiety along with uncertainty when they first walked into the hospital and saw the staff because they did not know what to expect regarding their treatment.

*“I was scared about what would happen inside. Will they even listen to me?”* (Male, 35 years old, medical unit.)

*“I had no idea where to go or what to do* when *I arrived.”* (Male, 50 years old, orthopedic unit.)

#### 3.1.2. Impact of Communication

A lack of proper communication on the part of the hospital staff was one of the most frequently mentioned issues. The participants expressed annoyance due to poor descriptions and a lack of clarity about their health conditions and treatment plans. The participants also mentioned the effect of staff attitude on emotional safety. The participants identified clear and compassionate communication by the health staff as an important factor that makes them feel safe.

*“The doctor just wrote something on paper and left without telling me what was happening.”* (Male, 42 years old, surgical unit.)

*“Whenever I asked questions, they either ignored me or told me to wait.”* (Female, 29 years old, medical unit.)

On the other hand, a few participants were satisfied with the level of communication with health staff.

*“The doctor explained my condition and treatment options in detail. I felt comfortable and informed.”* (Male, 40 years old, medical unit.)

#### 3.1.3. Emotional Stress Due to Treatment Delays

One key aspect of emotional suffering identified by the participants consisted of delays in receiving timely medical attention. The participants expressed increased dissatisfaction and anxiety resulting from extended waiting times for treatment, medication, and diagnostic interventions. They were concerned and reported feeling unsafe because of the lack of a proper mechanism for prioritizing their concerns.

*“I was in severe pain, but they just kept telling me to wait for my turn.”* (Male, 48 years old, orthopedic unit.)

*“Three days just for a test result, while I kept suffering. It was unbearable.”* (Female, 55 years old, medical unit.)

Conversely, some participants reported timely treatment and attention from medical staff.

*“I was admitted quickly, and the doctors started my treatment immediately.”* (Male, 38 years old, orthopedic unit.)

#### 3.1.4. Systemic Factors

One major source of emotional stress identified by the study participants consisted of systemic issues in the hospital. The patients complained about too much paperwork and ineffective management. The participants stated that these issues were challenging, particularly for patients with little literacy or a lack of family support. The lack of clear rules on hospital operations and pointless administrative obstacles caused further emotional suffering.

*“Why do I have to go to so many counters and fill so many forms just to get a simple approval?”* (Male, 60 years old, medical unit.)

*“They lost my file, and I had to redo my tests. It felt like nobody cared.”* (Female, 38 years old, medical unit.)

#### 3.1.5. Financial Factors

Financial issues such as the cost of treatment, insurance coverage, and unexpected hospital bills were the key concerns causing emotional distress for the participants. They became aware of hidden charges when discharged, adding to their emotional turmoil. To lessen the emotional weight related to hospital bills, the participants underlined the need for financial counselling and clear billing.

*“The treatment cost kept increasing, and I didn’t know what I was actually paying for.”* (Male, 44 years old, surgical unit.)

*“I was told insurance would cover everything, but at the end, I had to pay extra.”* (Male, 52 years old, medical unit.)

#### 3.1.6. Lack of Emotional Support

The participants revealed that they felt emotionally unsupported and isolated during their hospital stays. Many noted that they suffered emotionally because of a lack of psychological support, while some reported receiving emotional support from health staff. The patients wanted to receive more understanding care from the hospital staff, revealing the significance of staff attentiveness to and empathy for patients’ emotional safety.

*“Nobody asked how I was feeling”… “Days in the hospital feel endless with no one to talk to.”* (Male, 36 years old, orthopedic unit.)

*“I was crying in pain, but nobody tried to comfort me.”* (Female, 58 years old, medical unit.)

*“The doctor not only* treated *my illness but also gave me emotional support. That meant a lot.”* (Male, 52 years old, medical unit.)

#### 3.1.7. Post-Hospitalization Concerns

The participants stated that they received unclear directions and noted a lack of follow-up support when discharged from the hospital, leading to uncertainty and emotional stress. Many patients felt unsafe after leaving the hospital because of the absence of systematic discharge planning and follow-up care systems.

*“They just gave me a paper and said I could go home. I had no idea what to do next.”* (Male, 49 years old, medical unit.)

*“Who do I call if something goes wrong? Nobody told me.”* (Female, 41 years old, medical unit.)

### 3.2. Care Provider Perspectives

As shown in Table 2B, three exclusive non-overlapping themes emerged as a result of this thematic analysis of doctors’ and nurses’ perspectives regarding emotional safety and harm with respect to patients during hospitalization. The themes extracted from the data were as follows: factors contributing to emotional harm, coping mechanisms and solutions, and the impact of prior experiences and involvement.

#### 3.2.1. Theme 1: Factors Contributing to Emotional Harm

##### Systemic Factors

The participants reported that patients experience emotional harm during hospitalization because of certain environmental and institutional factors in the hospital environment, such as administrative inefficiencies, resource management, and poor hospital facilities. The participants believed that the main cause of their emotional suffering was the hospital system, which is mostly shaped by government regulations. In the local context, public hospitals are often under-resourced and overcrowded, resulting in negative effects on the emotional well-being of patients due to long waiting times and limited individualized attention. A lack of necessary resources, especially medical personnel, increases patient anxiety and emotional load, while a lack of available beds and a difficult admissions process produce delays that induce irritation and stress. Overloaded labs and testing facilities stretch wait times, hence adding to patient stress.

*“Patients are stress due to our admission process… and the admission counter, means that it is almost 800 m or 1 km away”* (Dr., 28 years old, medical unit)

*“… normally our tests take an hour or two, but some time the laboratory, it takes two to three hours”* (RN, 32 years old, obstetric unit)

*“Because these people have come to the hospital for the first time. And they do not know this place”* (RN, 25 years old, psychiatric unit)

##### Staff-Related Factors

The participants also mentioned that attitudes, actions, and inadequate hospital staff might emotionally harm patients. The participants’ answers show that the main elements influencing patient experiences include personnel shortages, overly demanding workloads, inadequate communication, and a lack of empathy. Some of the participants reported that a scarcity of staff compromised the quality of treatment; when staff are overburdened, they suffer from burnout, which can result in a lack of empathy towards patients.

Patients also suffer emotionally when staff members adopt harsh attitudes, dismiss their concerns, or fail to communicate effectively. The participants also revealed that patients find it challenging to understand their medical conditions or therapy since they struggle to successfully interact with personnel because of linguistic issues. According to the participants, patients are also stressed when they are not informed about their diagnoses or prognoses.

*“what I feel, which causes the most emotional harm, is that when you treat the patient, then sometimes I have seen that when you become overburdened, then automatically you have a lack of empathy”* (Dr., 34, surgical unit)

##### Patient-Related Factors

The participants also reported patient-related factors that can contribute to emotional harm. The participants’ answers show that patients’ emotional well-being during a hospital stay is largely shaped by personal stress, including financial hardship, a lack of medical knowledge, pre-existing anxiety, family support, and past trauma. They revealed that patients with financial difficulties are more worried and experience more emotional suffering when hospitalized. Additionally, patients who lack sufficient knowledge about their illness or treatment suffer more stress. The participants also revealed that patients who have had previous unfavorable hospital experiences carry emotional trauma that affects their present hospital stay and often results in fear, tension, and mistrust.

*“the anxiety level of a patient who comes to the hospital is much higher than before… When he comes to know that he has this problem, people do not get mentally prepared”* (Dr., 45, psychiatry)

*“Some patients worry about extra tests or procedures, thinking they are being done for financial gain rather than medical necessity.”* (RN, 25, surgical unit)

#### 3.2.2. Theme 2: Coping Mechanisms and Solutions

In regard to this theme, the participants identified certain solutions, such as improving communication and education, that can be used to minimize patients’ emotional harm during hospitalization, thereby improving communication and education, staff support and behavior, and hospital policy and systems.

##### Improving Communication and Education

The participants emphasized a need for honest, sympathetic communication and training in order to reduce emotional suffering during a hospital stay. The participants’ answers imply that efficient communication between patients and healthcare professionals can result in good emotional well-being and trust in the medical system. Counselling provides emotional support to patients so as to improve their treatment and recovery. The participants also revealed that delays and a lack of knowledge regarding medical processes cause discomfort; hence, prompt interventions and thorough explanations can help alleviate patient worries. Better patient education is clearly important since patients feel less stressed when they are familiar with their disease and the course of therapy. Patients feel ignored when doctors ignore their worries, so attentive listening can be employed to help build trust and engender emotional comfort. It is also imperative to design a simpler and more easily available system since a complex admission process exacerbates patient anxiety.

*…communication directly affect the patient. If there is a doctor and a patient comes to him, he does not listen to what the patient is saying or not…* (RN, 35, obstetric unit)

*“The patient should be explained in their local language”* (Dr., 28, medical unit)

##### Staff Support and Training

The participants reported a need for sufficient training and support for medical staff to provide compassionate care. The participants’ answers suggest that a lack of emotional support, personnel shortages, and poor training hinder patient treatment and exacerbate emotional suffering. The participants underlined the need for more doctors and nurses to ensure patients receive timely and sufficient treatment. Compassionate treatment is necessary since the way hospital staff treat patients greatly affects patients’ emotional well-being. The participants also noted that staff require training in managing the psychological and emotional needs of patients, emphasizing the need for their continuous education. Including psychologists in every ward is advised as a key intervention to assist with patient emotional management. Nurses are very important for patient care since their capacity to provide focused and encouraging treatment helps minimize emotional harm.

*“As long as our staff members are not educated, they don’t know how to deal with it”*

*“Psychologists should be there in each and every ward… So that they can deal with the patient on time”* (Dr., 45, psychiatry)

##### Hospital Policy and System Improvements

The participants emphasized the need to change the systems, policies, and practices of hospitals to foster more patient-centered and supportive surroundings. Hospitals should follow structured regulations to guarantee that patient admissions and treatment procedures cause minimal emotional harm. Some participants suggested that drugs and other hospital resources should always be available in order to prevent interruptions in patient treatment. Assigning hospital porters to help patients navigate the hospital process can help minimize uncertainty and discomfort. Offering patients choices for stress-relieving activities, including TV, films, or games, both physical and virtual, will help them manage emotional pain.

*“So I say that there should be a proper porter with them from ER to here. He should take them and explain to them that this is the process”*

*“And the procedure should be timely, there should be no delay in it. Because when the patient’s procedures are delayed, then their anxiety and tension level increases”* (RN, 40, Medical)

#### 3.2.3. Theme 3: Impact of Prior Experiences and Involvement

The third and final theme addresses how a patient’s emotional well-being during a hospital stay is influenced by their past experiences with healthcare and the degree of involvement in their treatment.

##### Influence of Previous Hospitalization

According to the participants, negative past experiences at a hospital induce anxiety, uncertainty, and discomfort during subsequent hospital visits, while positive events build confidence and engender comfort. Those who have had positive past experiences return to the hospital hopeful and confident that they will receive quality treatment. Patients with unfavorable past experiences, on the other hand, may be reluctant or afraid to be treated at the same institution. Patients struggling financially could be compelled to return to government hospitals even if they expect negative experiences, such as long wait times or inadequate treatment. The participants also mentioned that patients’ future perceptions of a hospital and their readiness to return to it largely depend on the behavior and attitude of the healthcare personnel during previous hospitalizations.

*“if he is admitted again, then he recalls all the things in his mind, what happened to him in the past experience, he thinks that I will go to such people who do not cooperate with him, they will harm him”* (RN, 35, Oncology)

##### Patient Involvement in Care and Decision-Making

The participants suggested it is necessary to actively involve patients in their treatment plans and decision-making procedures in order to improve their sense of control and reduce emotional harm. Patients who actively participate in their treatment feel more educated and in control, reducing their tension and anxiety. Encouraging patients to voice their concerns and participate in care decisions helps them maintain their dignity and improve their sense of safety.

*“Patients should also be aware of all these things, that what is the complication of this thing, and what is not… So, if he is a well-educated patient, then he will know more, that this thing is good for me, this is not. So, it will be easy for him to make a decision”* (Dr., 45, Psychiatry)

The results reveal that both providers and patients identified systemic issues as significant contributors to emotional harm in hospitals. The providers cited administrative inefficiencies and resource scarcity, while the patients noted confusing policies, excessive paperwork, and administrative obstacles. Staff-related factors, such as ill-mannered behavior, ignoring patients, and a lack of empathy, were also identified as negative by both patients and providers. The providers reported that patients experience financial hardship, which was echoed in the patient theme of financial burden. The impact of prior experiences was also acknowledged, as past negative experiences induced anxiety and mistrust.

The patients’ experiences illustrated the emotional suffering that the providers discussed, with themes such as anxiety upon admission, emotional stress due to treatment delays, and a lack of emotional support. The providers’ solutions addressed these challenges, emphasizing communication, education, staff support, and hospital policy improvements. This integrated perspective validates the factors of emotional harm identified by both sides, demonstrating that improving emotional safety requires multifaceted interventions targeting systemic, staffing, and patient-level factors.

## 4. Discussion

This qualitative study explored emotional safety and harm among hospitalized patients from the perspectives of patients and providers (doctors and nurses). Through a thematic analysis, this study highlights converging themes related to factors influencing emotional safety, coping mechanisms and solutions, and the overall impact on patients’ emotional well-being. This study underscores the idea that emotional safety is equally as critical as physical safety in patient care.

The findings from the perspectives of both patients and providers reveal several common factors contributing to emotional harm or feelings of unsafety among hospitalized patients. Both doctors/nurses and patients identified systemic issues. Doctors and nurses reported that hospital or system problems, administrative inefficiencies, and poor resource management contribute significantly to patient emotional suffering. These findings align with the results of other studies identifying the social environment of a ward and institutional competence as systemic factors affecting patients’ emotional well-being [13,14,15]. Patients reported emotional stress due to unclear hospital policies, excessive paperwork, and inadequate management. Complex systems can make patients feel that they must simply adjust or accept changes without consultation [16]. Staff highlighted that a scarcity of resources, particularly medical personnel and beds, increases patient anxiety and stress via provoking delays.

The attitudes and actions of staff were crucial factors from both viewpoints. Doctors and nurses noted that personnel shortages, overburdened staff experiencing burnout, harsh attitudes, dismissal of concerns, and poor communication contribute to emotional harm, as reported in previous studies [8,16,17]. Patients specifically reported feeling neglected, dismissed, and annoyed because of inadequate descriptions, a lack of clarity about their conditions, and perceived rude or dismissive behavior when seeking information [16]. Staff also noted linguistic issues hindering patients’ understanding of their conditions or therapies [18]. Patients wanted staff who were communicative, available, and trustworthy. The nature of interactions with providers impacts a patient’s sense of safety [13,14].

Our findings on the impact of systemic factors, communication issues, and staff-related factors resonate strongly with the patient-reported safety concerns identified by O’Hara et al. Their findings reveal that communication, staffing issues, and care environment problems are the most common patient safety concerns. This provided a unique perspective about safety that differs from and adds to the current definition of safety, often overlooked by traditional reporting systems, provides valuable insights into patient experiences, serves as an indicator for broader safety improvements, and emphasizes the importance of integrating the patient’s voice for a more comprehensive understanding of safety [4].

The patients, doctors, and nurses in this study noted that individual vulnerabilities and prior experiences, including financial hardship, a lack of medical knowledge, pre-existing anxiety, and past trauma, increase sensitivity to emotional harm [8].

The importance of ‘feeling safe’ in patients was also revealed by Sutton et al.’s research on abdominal surgery survivors. They highlighted the vulnerability patients experience, including uncertainty, serious health conditions, and dependence on others. They also highlighted epistemic injustice, where patients’ concerns are ignored, leading to power imbalances. Understanding patient safety requires valuing their experiences and addressing emotional and relational aspects of care [2].

Patients were adamant that financial concerns, including treatment costs, insurance coverage, and unexpected billing, constitute a key source of emotional distress. Transparent billing and financial counseling were identified as ways of reducing this stress and improving patient-centered care [12,17,18].

Patients also reported that anxiety upon entry into unfamiliar environments and a lack of knowledge about illnesses or treatment caused stress, aligning with studies on patient experiences and anxiety in hospitals [12]. Patients also specifically identified emotional stress due to treatment delays as a significant factor. This finding is supported by previous studies highlighting that timely services are essential for patients to feel safe, and delays compromise this sense of safety [1]. Patients reported feeling lonely and emotionally unsupported.

The perspectives of both the patients and providers emphasized the critical role of communication. Doctors and nurses highlighted honest, sympathetic communication; information sharing; empathetic listening; prompt responses; counseling; and patient education as vital strategies, as consistently reported in previous studies [1,13,18]. Previous research also highlighted the importance of communication in alleviating worries and providing emotional support. Educating patients reduces stress by improving their understanding of illness and treatment. Attentive listening builds trust, emotional support, and a sense of safety [1,9,13].

Both doctors and nurses emphasized the need for adequate resources, training, and emotional support for staff to allow them to provide compassionate care. They suggested continuous education and possibly including psychologists in wards to manage patients’ psychological needs. Nurses play a crucial role in providing focused treatment; similar findings have been reported in previous studies [12,16,19].

The providers suggested that hospital policies should prioritize patient-centered care and emotional well-being, with structured regulations for admissions and treatment. Previous research has suggested that readily available resources, simplified administrative processes, financial counseling, improved discharge planning, and stress-relieving activities can reduce uncertainty [3,14].

This study highlights the impact of past hospitalizations on patients’ anxiety and discomfort, with negative experiences leading to reluctance to return. Healthcare personnel’s behavior and attitudes during previous hospitalizations also influence patients’ future views.

This study may have several implications for practices, such as identifying the need for a patient safety paradigm that integrates the patient’s perspective. Both patient preparation and changes in care-provider practices are essential.

This study emphasizes the active engagement of patients in their care and decision-making to improve their sense of control and reduce emotional harm. Previous studies consistently emphasized the importance of patient involvement and active engagement for reducing emotional harm and fostering dignity and safety [3,14].

The factors identified in this study have tangible adverse effects on patients’ emotional health and overall perceptions of safety. When patients have little information, are met with inadequate communication, or feel overlooked and disregarded, the result can be significant emotional discomfort, encompassing dread, anxiety, guilt, uncertainty, and a sense of invalidation exacerbated by sensations of impotence and vulnerability, which may manifest physically, thereby prolonging recovery. Significantly, experiences of emotional distress can undermine patients’ trust in healthcare providers and the corresponding system [2,4].

Our findings highlight safety as an experiential and subjective phenomenon rooted in the feeling of feeling safe, emphasizing the need for a new patient safety paradigm. As Barrow’s Patients’ Safety Theory suggests, patients perceive safety as a subjective experience, often differing from objective measurable parameters prioritized in the clinical paradigm. Healthcare systems should value both being safe and feeling safe. Our study, comprehensively exploring emotional safety from both patient and provider perspectives, further substantiates Barrow’s argument that integrating the patient perspective requires broadening existing definitions and practices to genuinely reflect what truly matters to patients in their care [10].

Our findings also align with previous research that highlighted patient vulnerability and power imbalances in healthcare [2,10]. However, this study offers a novel contribution by placing emotional safety at the center of analysis, rather than addressing it as a secondary or supporting theme. Unlike earlier studies that briefly mention emotional aspects of care, we focus specifically on emotional safety as a distinct and important dimension of patient safety. A key strength of this study is the use of both patient and provider perspectives within the same resource-limited hospital setting. This approach allows us to identify shared and differing views on emotional safety and harm and to explore the systemic, interpersonal, and contextual factors that shape these experiences. These insights offer practical value for improving patient-centered care and can inform context-appropriate healthcare practices and policies.

For patients, we recommend enhancing emotional safety through preparation and empowerment, including through (1) educating patients and their families about the potential emotional consequences of safety events and medical errors, ideally through informative materials like brochures; (2) fostering patients’ active participation in their care planning and ensuring they receive relevant information to significantly enhance their sense of safety and security; (3) focusing on managing patient expectations by providing clear communication about procedures and treatment pathways to alleviate patients’ stress and bolster their sense of security; and (4) empowering patients to actively question unsafe practices or identify risky situations in order to foster genuine psychological safety, requiring an environment where patients’ concerns are heard and believed, free from fear of negative consequences or invalidation.

For providers, we recommend fostering emotional safety through comprehensive education, resources, and systemic changes, such as integrating psychological safety principles into daily practice and promoting empathy, kindness, respect, and attentive listening. Furthermore, addressing provider burnout, establishing systems for recognizing and analyzing disrespect and emotional harm, fostering an inclusive atmosphere, and ensuring fair responses are given to address reported emotional harm may also be helpful in reducing patients’ emotional harm and ultimately improving emotional safety.

Further studies using different methodologies, such as interviewing patients within the same care units, are required in order to address the homogeneity of factors affecting emotional safety. Quantitative validation of emotional harm factors could determine their frequency and impact on outcomes. An evaluation of the effectiveness of interventions designed to support emotional safety is also needed. Cross-hospital comparisons could identify effective practices and guide targeted interventions in diverse healthcare environments. Exploring emotional safety from the perspectives of other stakeholders is also recommended for future research.

### Limitations

The limitations of this study may include its small sample sizes and the specific hospital environments and cultures analyzed. We also acknowledge that FGDs with patients can introduce limitations, particularly with respect to the potential for social desirability bias or silence regarding sensitive issues. Furthermore, the recruitment of patients from various units may have introduced variability in the results. We also acknowledge that there is some conceptual overlap between emotional dissatisfaction and emotional safety in our findings. Future studies may also explore the distinctions between these two concepts in more depth. Furthermore, we also acknowledge the underrepresentation of quotes from female patients. It is possible that gender dynamics, comfort in group settings, or cultural factors influenced female patients’ willingness to speak, which may have affected the balance of perspectives captured. This study did not explicitly explore the patients’ preferences or capacity for involvement in care and decision-making. As a result, insights into patient involvement were only gathered from healthcare providers. Future research should directly examine patients’ views on their participation, including potential variations in willingness or ability to engage.

## 5. Conclusions

This qualitative study exploring the perspectives of healthcare providers and patients provides a comprehensive understanding of emotional safety and harm in hospital settings. The findings underscore the complex interplay of systemic, staff-related, and patient-specific factors that contribute to emotional harm. Addressing emotional safety greatly depends on improving communication, providing adequate staff support and training, simplifying administrative processes, offering financial counseling, integrating emotional support into care, and improving discharge planning and follow-ups. Implementing practical strategies identified by providers can help promote emotional safety and minimize emotional harm. By incorporating the insights from both the givers and receivers of care, healthcare institutions can work towards creating a more patient-centered environment that supports patient emotional well-being, enhances patient outcomes, and builds trust.

## Figures and Tables

**Table 1:** (**A**) Demographics of the study participants (patients). (**B**) Demographics of study participants—providers (doctors and nurses).

(**A**)		
**S/NO**	**Category**	**No.**
	**Age in years**	
	18–40	10
	41–60	10
	60 and above	5
	Gender	
	Male	20
	Female	5
	Education	
	Primary	4
	Secondary	7
	Graduate and above	14
	Unit of admission	
	Medical	10
	Surgical	5
	Orthopedic	10
	Hospital stay	
	1 week	7
	2 weeks	12
	More than 2 weeks	5
(**B**)		
**S/NO**	**Characteristics**	**Number**
	**Age in years**	
	25–30	8
	31–40	4
	41–50	3
	**Gender**	
	Male	9
	Female	6
	**Profession**	
	Doctor	7
	Nurse	8
	**Working Unit**	
	Medical	3
	Surgical	3
	Obstetrics	3
	Oncology	3
	Psychiatry	3
	**Experience in years**	
	1–5	6
	6–10	6
	More than 10	3

**Table 2:** (**A**) Thematic analysis of the data collected from patients through focus group meetings. (**B**) Thematic analysis of the data collected from providers (doctors and nurses) through interviews.

(**A**)				
**S/NO**	**Themes**	**Category**	**Codes**	**Quotes**
1	Anxiety upon Admission	Pre-Hospital Anxiety	Fear and uncertainty upon hospital entry	*“When we come to the hospital, our initial feeling is more about how we will be treated and dealt with, rather than the illness itself.”* (Male, 35, Medical.)
			Stress due to an unfamiliar hospital environment	*“I had to go to the hospital again and again to get treatment… It is sad that I cannot go back home* (Female, 55, Medical)
2	Impact of Communication	Lack of Clear Information	Unclear explanations from hospital staff	*“Doctors prescribe medicine without explaining it properly. Patients have to repeatedly ask for explanations”* *“We expect emergency treatment after being admitted, but that often gets delayed. No one explains why.”* (Male, 48, Orthopedic)
		Staff Attitude and Responsiveness	Emotional distress caused by dismissive behavior	*“I kept on calling for help, but the doctors did not listen to me”* (Male, 35, Medical.)
			Perceived rude behavior of medical personnel	*“Some people behave arrogantly when given a little authority.”* (Male, 42, surgical.)
3	Emotional Stress due to Delayed Treatment	Waiting for treatment	Anxiety due to treatment delays	*“There were vacant beds, but in their online system the beds were shown as occupied… This made me so disturbed.”* (Male, 38, orthopedic.)
			Emotional distress due to delayed pain management	*I was in pain on a stretcher, and I expected immediate relief… but no treatment was given after a long time.”* (Male, 50, orthopedic)
4	Systemic Factors	Administrative Inefficiencies	Stress due to unclear hospital policies	*“There should be regular surveys or interviews with patients to ensure policies are being implemented.”* (Male, 52, medical) *“Policies are well-formed but need proper implementation. Decisions made in meetings should be followed up.”* (Male, 42, surgical)
5	Financial Burdens	Cost of Treatment	Anxiety due to high treatment expenses	*“We are poor people but we are worried about our health.* (Female, 55, Medical)
			Stress due to unclear billing	*“They just keep writing something on paper, and…don’t know if it’s a bill…* *“They keep adding charges… How can I afford this?”* (Female, 38, medical.)
(**B**)				
**S/No**	**Themes**	**Categories**	**Codes**	**Quotes**
1	Factors Contributing to Emotional Harm	Systemic Factors	Public hospital setup	*“On the government setup, the emotional stress of the patient or his relatives is mostly high… because the patient’s rights are not considered properly.”* (Dr., 45, psychiatry)
			Bed allocation problems	*“When the patient comes for admission, he has the biggest problem of bed allocation…”* (RN, 40, Medical)
		Staff-Related Factors	Ill-mannered behavior	*“patients will say that the staff’s behavior is not right… they are afraid to ask me.”* (RN, 35, Oncology)
			Lack of empathy	*“There should be a receptionist and PRO (Public Relations Officer) who should support the patient’s family… but no one listens to his complaints.* (Dr., 30, Surgical)
		Patient-Related Factors	Financial stress	*“They do not have money, nor do they have a health care… this is also a factor for their emotional harm* (Dr., 28, medical).
			Past experiences	*“He is already scared… someone told him from the past that the staff is ill-mannered.”* (Dr 30, Surgical)
2	Coping Mechanisms and Solutions	Improving Communication and Education	Proper counseling	*“At the arrival and on hospital admission… we explain everything to patient and their attendant* (RN, 40, Medical)
			Timely procedures	*“Sometimes we order CT or MRI, but they give us time 10 days later… this exhausts the patient and attendant emotionally.”* (Dr., 45, Psychiatry)
		Staff Support and Training	Empathetic behavior	*“I try to help those who don’t have availability of a health card…I understand their concerns* (RN, 35, Oncology)
			Training of staff	*“Proper communication skills should be in our staff and doctors… so the emotional distress of the patient can be controlled* (Dr., 45, psychiatry)
		Hospital Policy and System Improvements	Policies designed to reduce emotional harm	*“If there is a proper triage department… then many problems causing emotional distress can be reduced and the patient can be facilitated.”* (Dr., 28, medical)
			Relaxing environment	*“The environment of the hospital should be proper. There should be a reception… to guide patients on which ward, which floor.”* (Dr., 34, surgical unit)
3	Impact of Prior Experiences and Involvement	Influence of Previous Hospitalization	Good interaction benefits	*“If they had a good experience… they would request the same doctor or nurse who previously took care of them.”* (RN, 35, obstetric unit)
		Patient Involvement in Care and Decision Making	Patient involvement	*“We involve patients and their attendants in the care process, which helps in reducing their stress.”* (Dr., 28, medical)

## Data Availability

Data are available upon reasonable request from the corresponding author.

## Supplementary Materials

The following supporting information can be downloaded at: https://www.mdpi.com/article/10.3390/healthcare13151842/s1, Interview Guided.
