# Supplementary material for: Exploring Emotional Safety and Harm Among Hospitalized Patients: A Qualitative Study of Patients’ and Providers’ Perspectives
Source: Healthcare (Basel). 2025 Jul 29;13(15):1842. doi: 10.3390/healthcare13151842 (PMC12346503; doi:10.3390/healthcare13151842)
Supplement: Supplementary file 1 [file healthcare-13-01842-s001.zip › healthcare-3680636-supplementary.pdf]

FACTORS AFFECTING EMOTIONAL SAFETY OF PATIENTS

(Questions to ask from doctors and nurses)

1. What do you think about patients' experiences of emotional harm while being hospitalized?
2. What is your perception of the factors that may contribute to emotional harm to patients in the hospital setting?
3. According to your perception how patients cope with their emotional distress while being hospitalized?
4. What are the staff members' attitudes towards patients' emotional needs and how do they address them?
5. How can hospital policies and procedures be modified to prevent or mitigate emotional harm to patients?
6. How do patients' prior experiences with healthcare impact their emotional well-being while being hospitalized?
7. What types of emotional support or resources do patients find helpful while being hospitalized?
8. How can patients be more involved in their care and decision-making to prevent or minimize emotional harm during their hospital stay.

<u>DETERMINING PATIENT SAFETY DURING HOSPIATLIZATION</u>

<u>(Questions to ask from patients)</u>

1. What you think about your emotional safety during hospitalization?
2. Can you explain that how your voice/ concern is listened and addressed by doctors/ nurses when you are hospitalized?
3. What particular aspects of hospitalization makes you feel emotionally unsafe or uncomfortable?
4. How do you think about the level of privacy and confidentiality you receive during your hospital stay?
5. What do you think about your access to emotional support and counseling during your hospitalization?
6. How do you feel about the staff's ability to communicate effectively and empathetically with you throughout your hospitalization?
7. Are there any changes or improvements you would suggest to ensure that patients feel emotionally safe and supported during hospitalization?
